# Transcriptome analysis of the procession from chronic pancreatitis to pancreatic cancer and metastatic pancreatic cancer

**DOI:** 10.1038/s41598-021-83015-4

**Published:** 2021-02-09

**Authors:** Jihao Tu, Zhehao Huang, Yin Wang, Meijing Wang, Zukun Yin, Xianglin Mei, Meiying Li, Lisha Li

**Affiliations:** 1 The Key Laboratory of Pathobiology, Ministry of Education, College of Basic Medical Sciences, Jilin University, Changchun, China; 2 Department of Neurosurgery, China-Japan Union Hospital of Jilin University, Changchun, China; 3 Department of Otorhinolaryngology-Head and Neck Surgery, Minda Hospital of Hubei Minzu University, Enshi, China; 4 Department of Pathology, The Second Hospital of Jilin University, Changchun, China

**Keywords:** Cancer, Metastasis, Tumour biomarkers

## Abstract

Exploring the underlying mechanisms of cancer development is useful for cancer treatment. In this paper, we analyzed the transcriptome profiles from the human normal pancreas, pancreatitis, pancreatic cancer and metastatic pancreatic cancer to study the intricate associations among pancreatic cancer progression. We clustered the transcriptome data, and analyzed the differential expressed genes. WGCNA was applied to construct co-expression networks and detect important modules. Importantly we selected the module in a different way. As the pancreatic disease deteriorates, the number of differentially expressed genes increases. The gene networks of T cells and interferon are upregulated in stages. In conclusion, the network-based study provides gradually activated gene networks in the disease progression of pancreatitis, pancreatic cancer, and metastatic pancreatic cancer. It may contribute to the rational design of anti-cancer drugs.

## Introduction

Pancreatic cancer, comprising mostly pancreatic ductal adenocarcinoma (PDAC), is an extremely lethal disease. The disease frequently causes few symptoms before it develops to the advanced stage. Those who do develop symptoms often have non-specific complaints^1,2^. Multidetector CT angiography, MRI and Endoscopic ultrasound are the recommended initial imaging technique for accurate and timely diagnosis^3–5^. CA19-9, a validated serum biomarker, maintains a sensitivity of 79–81% and specificity of 82–90% for the diagnosis of the PDAC in symptomatic patients^6^. Patients who can undergo surgical resection had 5-year survival rates of 10–25%. For patients who cannot be surgically removed, effective systemic treatment provides a median overall survival of 18.8 months^7^. The current standard of care for advanced or metastatic PDAC provides only months of overall survival benefit^8^. For patients with PDAC, more effective therapy is urgently needed.

The gene networks of pancreatic cancer are very complex, such as KRAS and KRAS downstream effectors^9^, Yap-Myc-Sox2-p53 regulatory network^10^, miR-665/TGFBR1-TGFBR2/SMAD2/3 pathway^11^, and highly dynamic tumor microenvironment (TME)^12^. The non-cancer cell compartment of a tumor including a variety of resident and infiltrating host cells, secreted factors and extracellular matrix proteins, is collectively known as the TME. In pancreatic cancer, TME is highly immunosuppressive and fibrotic^13^ and composed largely of regulatory T-cells (Tregs), myeloid-derived suppressor cells (MDSCs), and other immunosuppressive cells^8^. Most cellular components exert their functions through complicated biological networks^14^. The study of gene networks can give us an understanding of complex systems^15,16^. Gene co-expression network analysis reveals the typical characteristics of prognostic genes. Prognostic genes were enriched in modules^17^.

A lot of research has analyzed the difference between pancreatic cancer and normal state, but little research has yet shown the progress of pancreatic disease. Most work makes simple comparisons between the two groups or picks the module with the highest correlation coefficient with cancer. Here we put the normal, chronic pancreatitis, PDAC and metastatic disease groups together in the WGCNA analysis. Moreover, we selected modules with changing correlation coefficients in the four states. This is a unique aspect of this research. Here we consider the different stages of pancreatic disease as different traits and assume that these four traits are continuous. Then calculate the correlation between the module and these traits. If some genes are gradually upregulated among the normal pancreas, pancreatitis, pancreatic cancer and metastatic pancreatic cancer, they are likely to be classified in the same module. These specific modules will show a gradually increasing correlation of traits from negative to positive. Therefore, we constructed pancreatic disease progression networks at the transcriptional level.

## Methods

### Gene expression data and data analysis

Gene expression profiling data E-EMBL-6 were obtained from the European Bioinformatics Institute database. We list the detailed information of the samples (www.ebi.ac.uk/arrayexpress, Table S1). Gene expression profiling data E-EMBL-6 contains the stages of the normal state, chronic pancreatitis, pancreatic cancer, and metastatic pancreatic cancer, each with nine samples^18^. Nine chronic pancreatic tissue samples were obtained from two female and seven male patients (median age 52 years; range 42–62 years). Nine pancreatic cancer tissue samples were obtained from seven male and two female patients (median age 63 years; range 53–77 years). Nine metastatic pancreatic cancer tissue samples were obtained from four female and five male patients (median age 58.5 years; range 58–78 years). All individuals had histologically confirmed pancreatic ductal adenocarcinoma. In addition, normal human tissue samples were obtained through an organ donor program from nine previously healthy individuals (five male donors, four female donors; median age 55 years; range 21–73 years). Freshly removed tissue samples were snap-frozen in liquid nitrogen immediately on surgical removal and maintained at − 80 °C until use. Data annotation is based on the Unigene Build #172 (www.ncbi.nlm.nih.gov/entrez/query.fcgi?db=UniGene). To analyze the expression signature, multiple supervised and nonsupervised clustering, as well as statistic methods, were applied by the open-source Java-based software package Multiexperiment Viewer version 4.9.0 (MeV, https://sourceforge.net/projects/mev-tm4/files/mev-tm4/). All clustering methods included hierarchical clustering, K-Means clustering, and Self-organizing maps. Based on these data, significance analysis of microarrays (SAM) was performed to select the inversely regulated genes. Differentially expressed genes (DEGs) were obtained by fold change. The |log2FoldChange| > 1 was set as the cutoff criterion.

### FunRich site for expression analysis of DEGs

FunRich is stand-alone software used primarily for functional enrichment and interaction network analysis of genes and proteins. FunRich is designed to handle a variety of gene/protein data sets irrespective of the organism. Additionally, users have more than 13,320 different background database options. DEGs were enriched by biological processes, cellular components, molecular function, biological pathway and the site of expression analysis of the software, respectively. The top ten items of DEGs were ranked according to − log10 (P-value). The comparison between total upregulated and downregulated DEGs was done based on the percentage of DEGs. The test P-value of less than 0.05 was considered significant.

### Weighted gene co-expression network analysis

Weighted gene co-expression network analysis (WGCNA) is a method to calculate correlated gene expression in the form of adjacency matrices (networks)^19^. We used WGCNA to analyze a total of 9046 gene expressions from EMBL-6. The modules were detected by hierarchical clustering. So, genes with similar expression patterns will be classified into the same module. The first principal components of each module were summarized as the module eigengene. Then the associations between the modules or rather the module eigengene and the pancreatic pathological process can be estimated. In the sample phenotypic data, we set a total of four columns, which are normal tissue, chronic pancreatitis, pancreatic cancer, and metastatic pancreatic cancer, corresponding to four types of samples. From this, we convert the sample phenotypic data into a 0–1 matrix. Then, calculate the correlation coefficients between the module eigengenes and the traits. Among these modules, some showed a negative correlation with normal pancreatic tissue and a larger and larger correlation with the three kinds of diseased tissue. Such modules are considered closely related to pathological processes, and so do the genes in the modules. We tested these genes for enrichment in Gene Ontology (GO) biological processes with functions implemented in clusterProfiler R package^20^. In WGCNA, the degree to which the expression of one gene change with the expression of another gene is quantified as connectivity. It constructs a scale-free network so almost all the connectivity will be kept. The difference between the connectivity can be amplified by power operations. After multiple power operations, the connectivity with a lower value will approach 0, and the connectivity with a higher value will receive less impact. WGCNA calculate the connectivity between each gene and the module eigengenes. From each of the concerned modules, we select twenty genes with the highest weighted-connectivity. Then we analysis them with the informatic tool STRING, a database of known and predicted protein–protein interactions^21^ and tool COEXPEDIA which explores biomedical hypotheses via co-expression associated with medical subject headings^22^. The result is represented by a graph drawn by Cytoscape^23^. We used GSE15471, GSE62452, GSE56560, GSE42952, TCGA-PAAD, TCGA-LUAD, The Human Protein Atlas (HPA), and R package hpar^24,25^ to validate our results.

### Ethical approval

This article does not contain any studies with human participants or animals performed by any of the authors.

## Result

### Chronic pancreatitis, pancreatic cancer, and metastatic pancreatic cancer have distinct gene signatures

**Figure 1:**
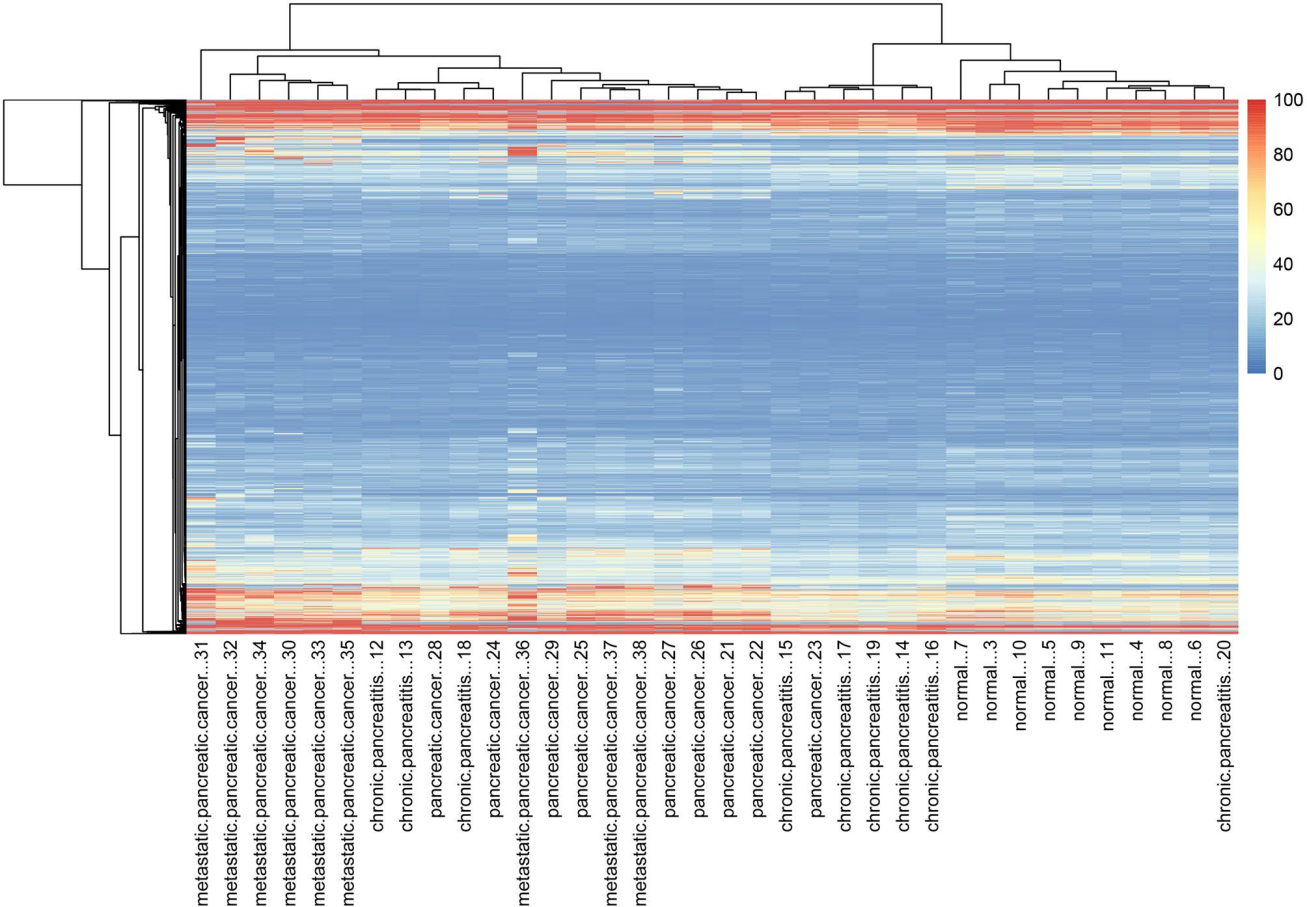
The cluster of all the genes in 36 samples including normal state, chronic pancreatitis, pancreatic cancer, and metastatic pancreatic cancer, each with nine samples.

**Figure 2:**
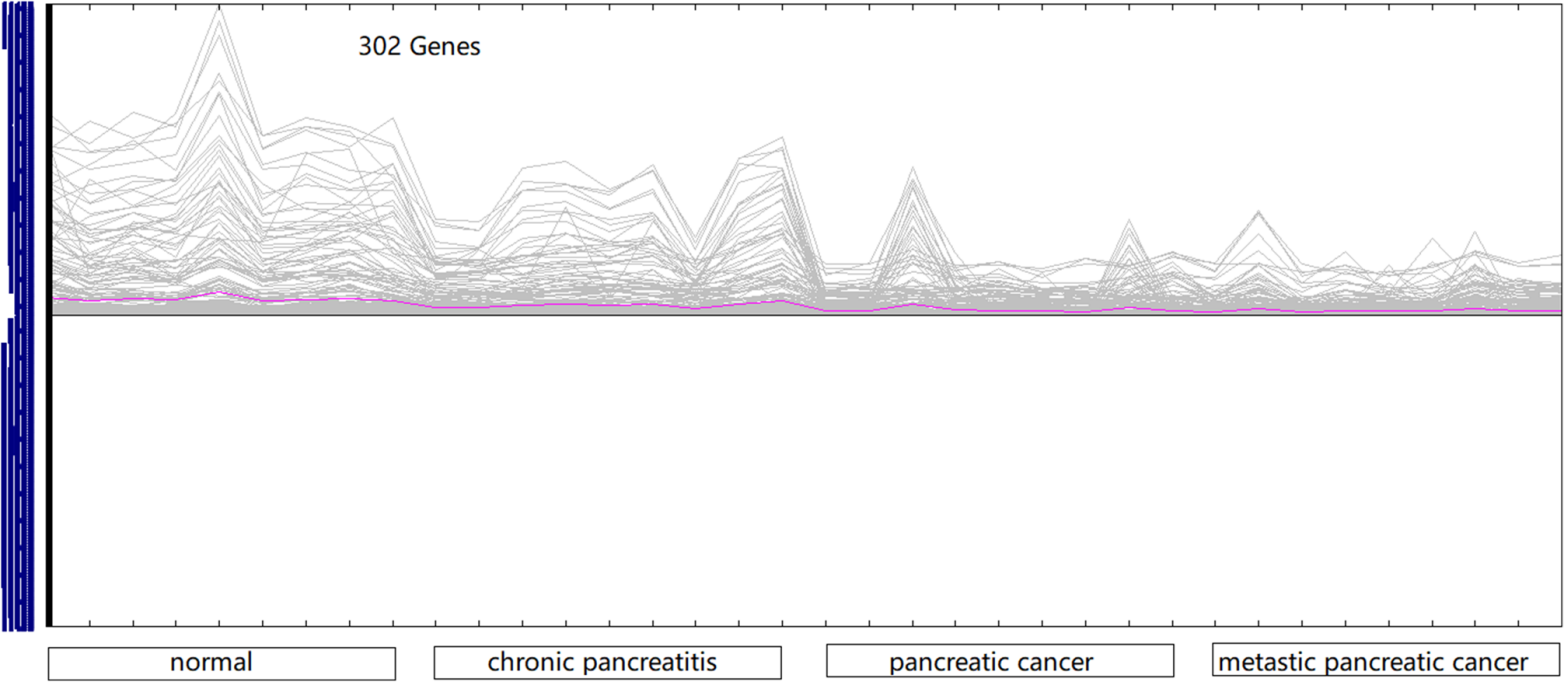
The expression graph of significant DEGs by SAM.

A total of 9046 gene expressions were obtained, and all the samples were clustered according to the gene expression value (Fig. 1, Table S2). Chronic pancreatitis and pancreatic cancer could not be distinguished very well in the cluster map. Probably pancreatitis shares some common gene expression characteristics with pancreatic cancer. By significance analysis of microarrays provided by MeV, 302 statistically significant DEGs were identified (Fig. 2).

There are 965 DEGs in chronic pancreatitis (Table S3), 2060 DEGs in pancreatic cancer (Table S4) and 2517 DEGs in metastatic pancreatic cancer (Table S5) with |log2FoldChange| > 1 set as the cutoff criterion. As pancreatic cancer progress, more and more genes are differentially expressed. The number of DEGs was the largest in metastatic pancreatic cancer. There are 302 DEGs by SAM method (Table S6).

### GO term enrichment analysis

**Figure 3:**
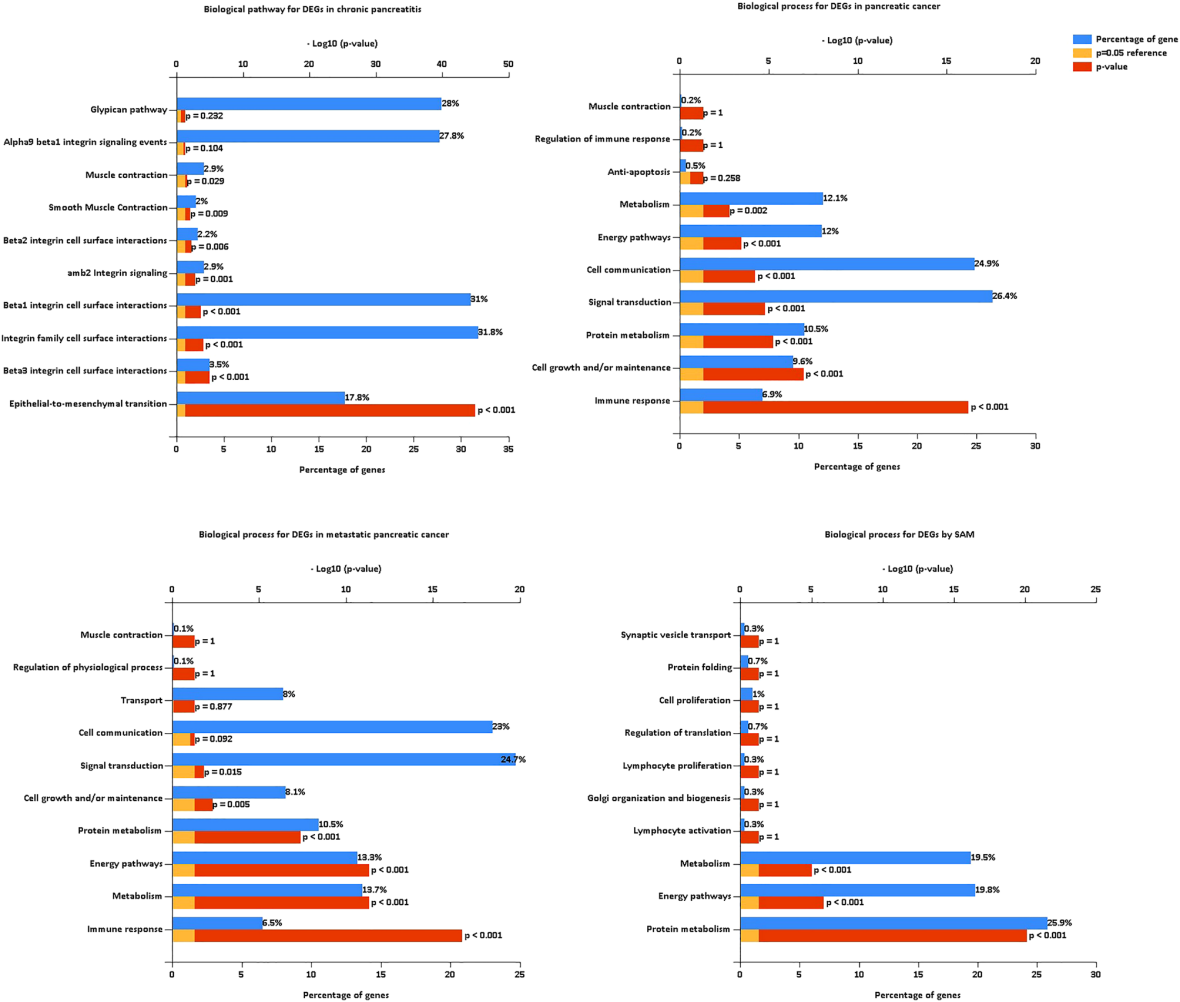
GO biological process for DEGs of chronic pancreatitis, pancreatic cancer, metastatic pancreatic cancer, and SAM, respectively.

**Figure 4:**
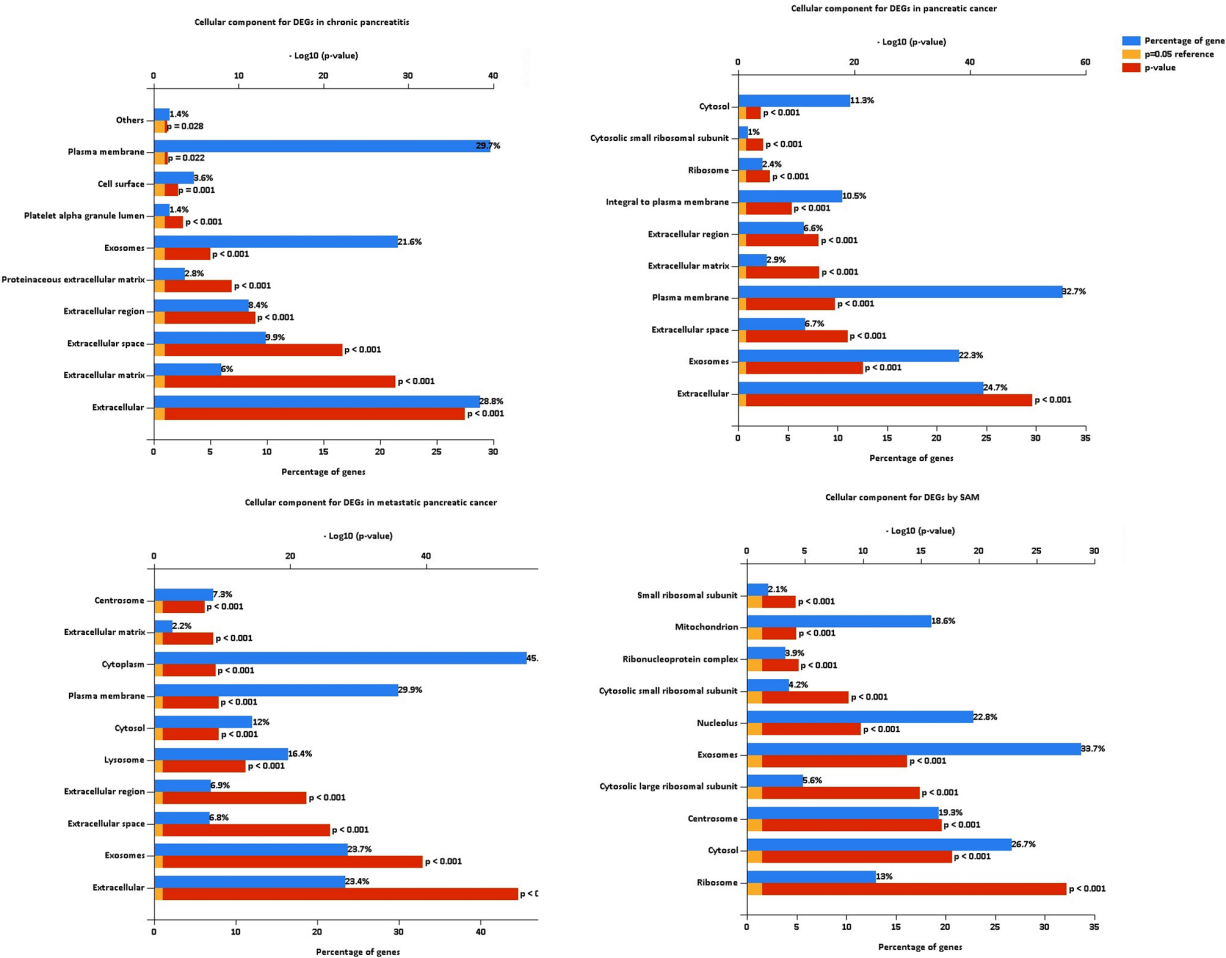
GO cell component for DEGs of chronic pancreatitis, pancreatic cancer, metastatic pancreatic cancer, and SAM, respectively.

**Figure 5:**
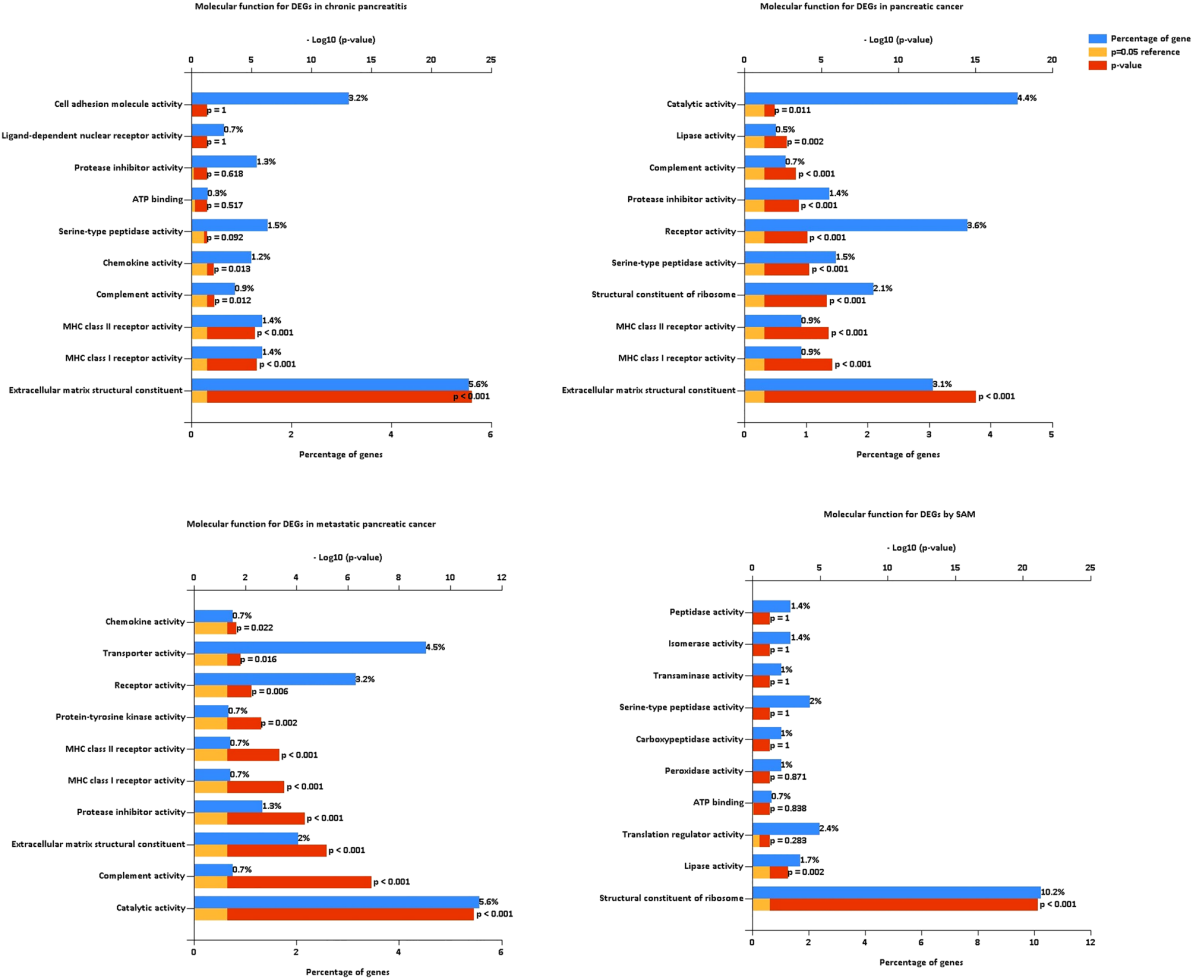
GO molecule function for DEGs of chronic pancreatitis, pancreatic cancer, metastatic pancreatic cancer, and SAM, respectively.

Cell growth and maintenance change in all the pancreatic cancer progression. Immune response changes in pancreatic cancer and metastatic cancer. The GO biological process analysis of chronic pancreatitis DEGs was enriched in cell growth and maintenance (Fig. 3, Table S3); the pancreatic cancer DEGs were enriched in immune response, cell growth and maintenance, protein metabolism, etc. (Fig. 3, Table S4); the metastatic pancreatic cancer DEGs were enriched in immune response, metabolism, energy pathway, protein metabolism and cell growth and maintenance (Fig. 3, Table S5). The SAM DEGs were enriched in lymphocyte activation, lymphocyte proliferation, protein metabolism, energy pathway and metabolism (Fig. 3, Table S6). The GO cellular component analysis of DEGs in all the pancreatic cancer progression was significantly enriched in extracellular matrix/region/space, exosomes, and plasma membrane (Fig. 4, Tables S3, S4, and S5). The SAM DEGs were enriched in ribosome, cytosol, etc. (Fig. 4, Table S6). The GO molecular function analysis of DEGs in all the pancreatic cancer progression was significantly enriched in extracellular matrix structural constituent, MHC class I receptor activity, and MHC class II receptor activity (Fig. 5, Tables S3, S4, and S5). The SAM DEGs were enriched in the structural constituent of ribosome and lipase activity (Fig. 5, Table S6).

### Site of expression analysis

**Figure 6:**
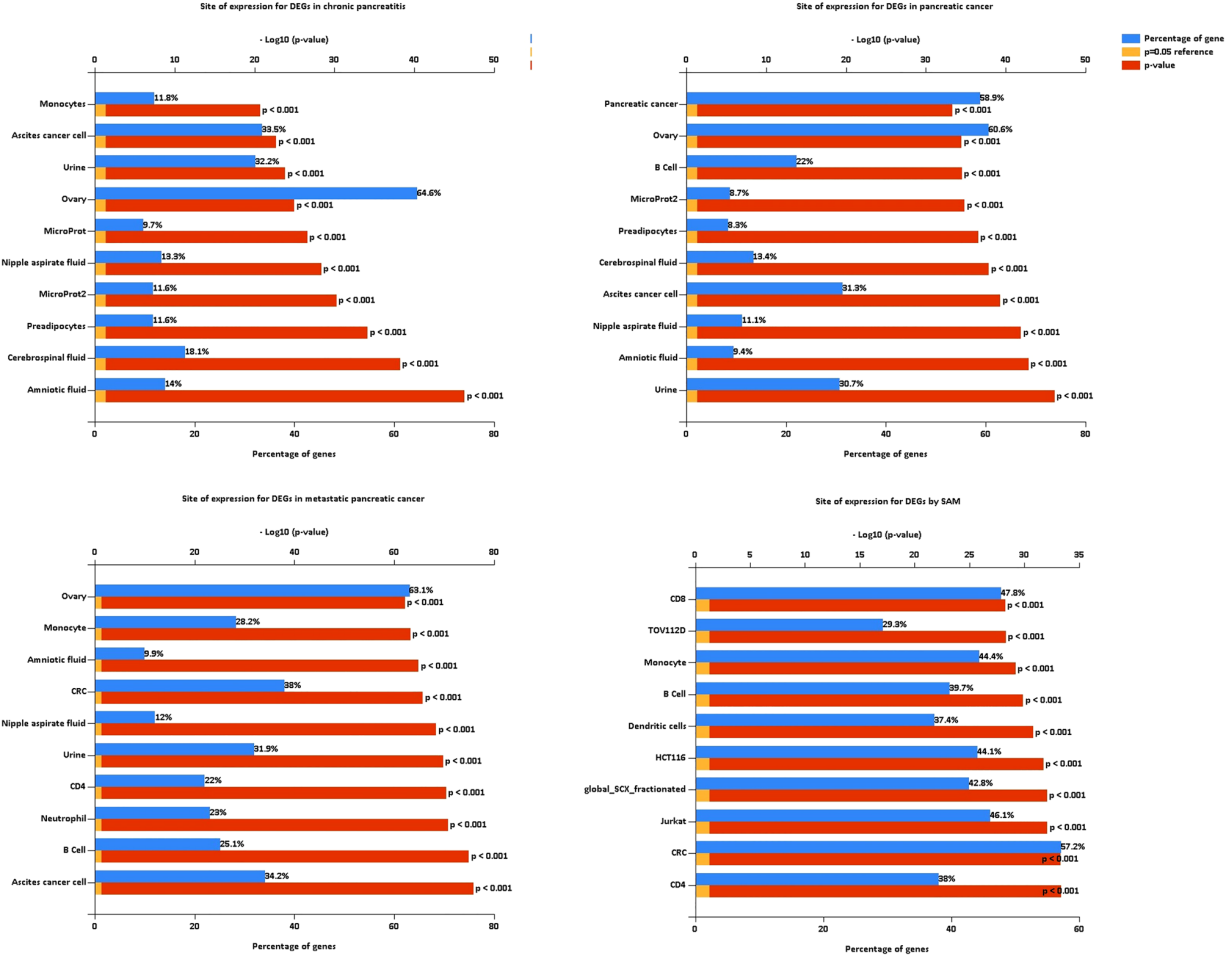
FunRich site of expression analysis for DEGs of chronic pancreatitis, pancreatic cancer, metastatic pancreatic cancer, and SAM, respectively.

The site of expression of DEGs in all the pancreatic cancer progression was significantly enriched in fluid and urine (Fig. 6, Tables S3, S4, and S5). The site of expression was further analyzed in Fig. 6. We obtained the gene rankings based on the percentage of DEGs after FunRich software analysis and exported the top ten items. The output pictures were automatically ranked according to − log10 (P-value). Immune cells emerged in a multitude only in metastatic pancreatic cancer. The SAM DEGs were enriched in many immune cells, including CD4 T cells, CD8 T cells, monocyte, dendritic cells, B cell, etc. (Fig. 6, Table S6).

### Biological pathway analysis

**Figure 7:**
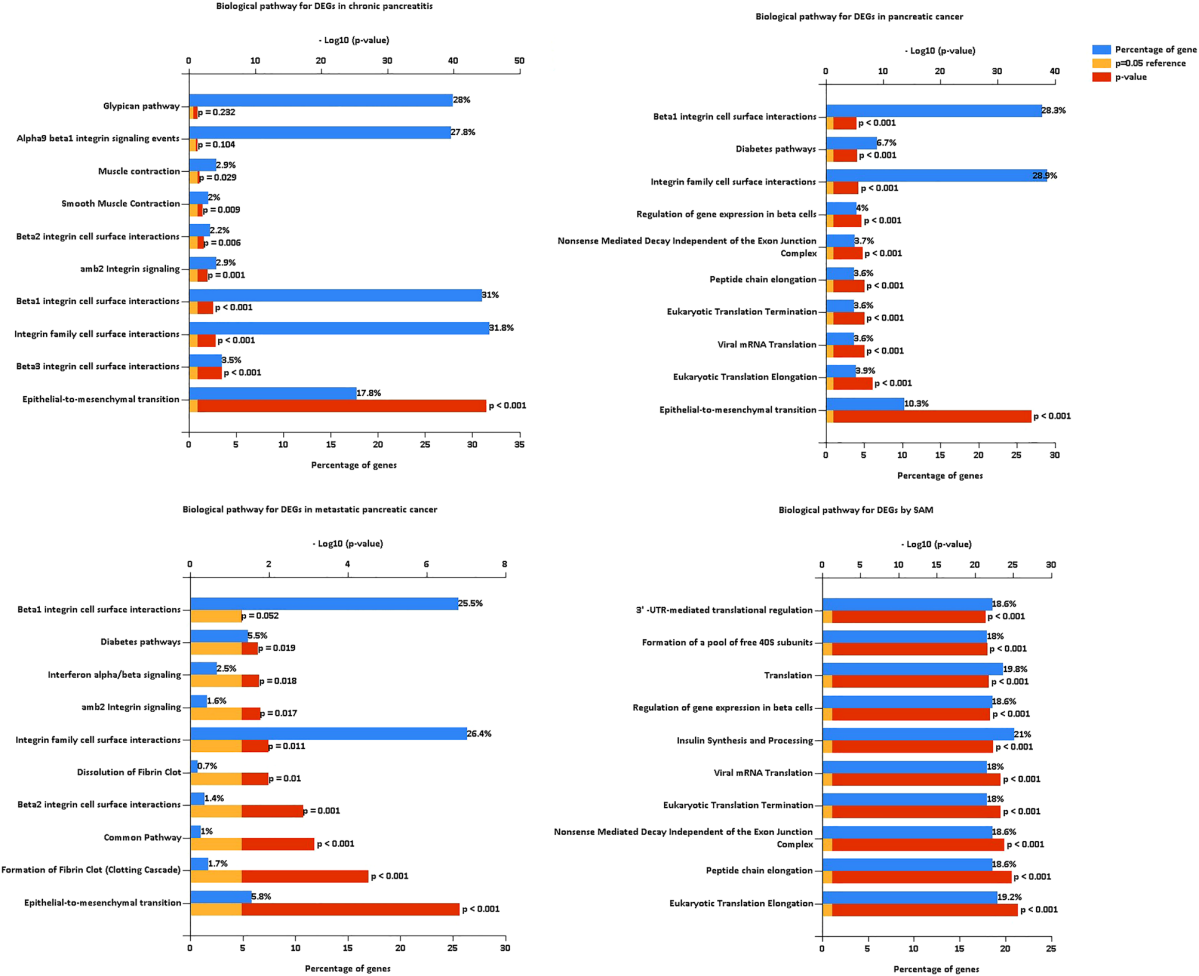
Biological pathway analysis for DEGs of chronic pancreatitis, pancreatic cancer, metastatic pancreatic cancer, and SAM, respectively.

The biological pathways of DEGs in all the stages of pancreatic cancer progression were significantly enriched in epithelial-to-mesenchymal transition and integrin family cell surface interaction (Fig. 7, Tables S3, S4, and S5). The DEGs of chronic pancreatitis were enriched in integrin family cell surface interaction (beta1, beta2, beta3) (Fig. 7, Table S3). The DEGs of pancreatic cancer were enriched in translational elongation and termination to support new protein synthesis (Fig. 7, Table S4). The SAM DEGs were enriched in all the levels of gene expression regulation, including mRNA, protein, etc. (Fig. 7, Table S6).

### Weighted gene co-expression network analysis

After network construction, 19 gene co-expression modules were identified. The number of genes in the modules varies between 47 and 1233 (N = 9,046, 758 genes were “gray” genes, not assigned to a module) (Table S7). We selected the best soft-thresholding β = 6 to ensure a scale-free network. Other details of the WGCNA analysis were reported in the supplement (Supplementary Figs. S1, S2, S3 and S4).

**Figure 8:**
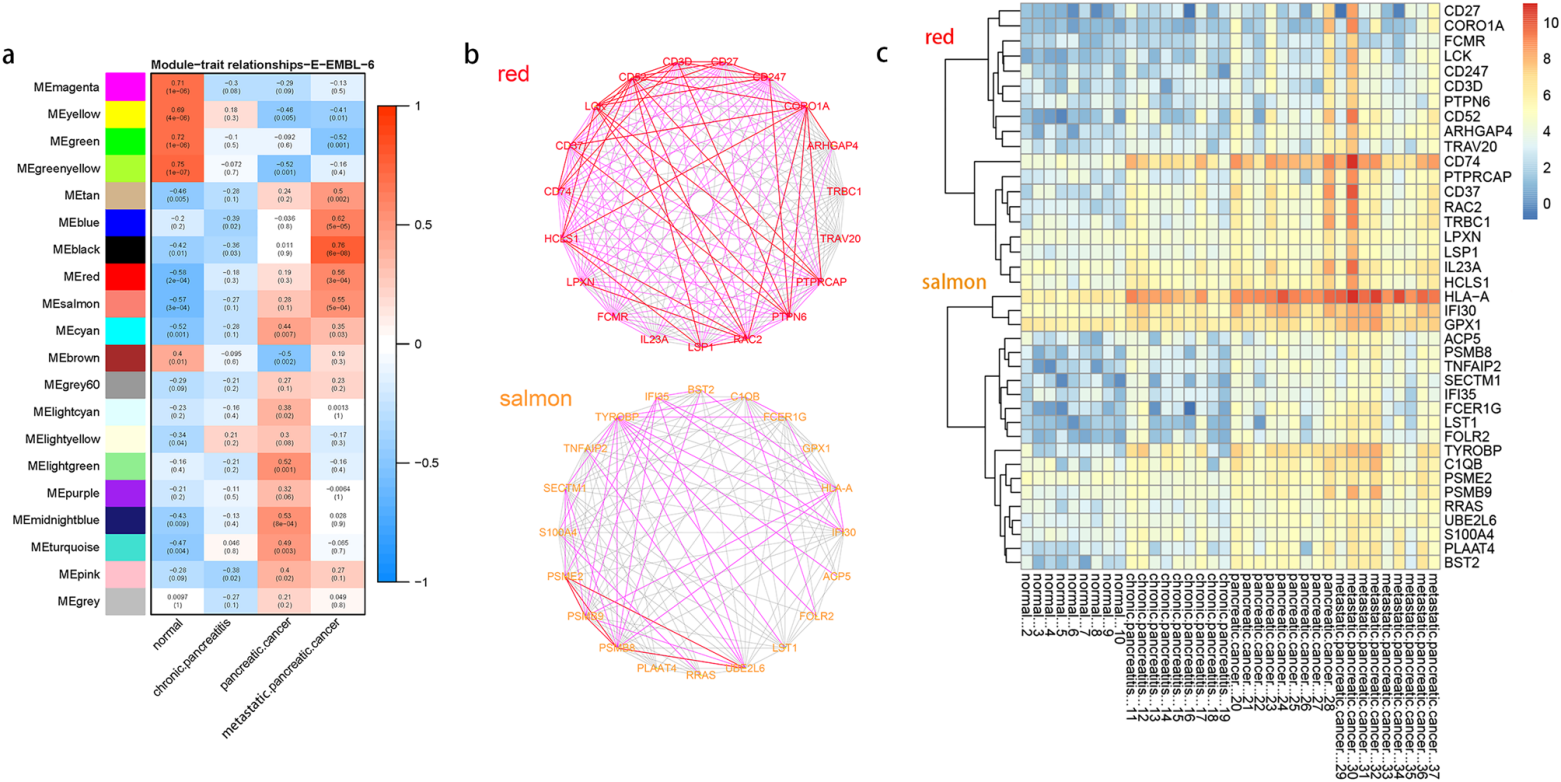
Potential pancreatic disease-related genes selected by WGCNA and further analysis. (**a**) Modules correlated with normal and diseased pancreatic tissue. (**b**) The verification of co-expression of the top20 genes in red and salmon modules in STRING and coexpedia. The verification result is represented by a graph drawn by Cytoscape. The dark lines show the co-expression in this research. The pink lines show the co-expression verified by STRING or coexpedia at the same time. The red line shows the co-expression verified by STRING and coexpedia. (**c**) The heatmap of top20 genes in red and salmon module. The expression has been converted by log2 (x + 1).

We selected modules significant for association with the pancreatic pathological process and performed further analysis (Fig. 8). In the module-trait relationship obtained, several modules got raising correlation coefficients from the normal state to metastatic pancreatic cancer (Fig. 8a). The correlation coefficients of these modules vary from negative to positive. They are red, salmon, tan, and black modules. GO biological process analysis shows genes in the red module most enriched in T-cell activation, in lipopolysaccharide and interferon in the salmon module, in protein process and gene silencing in the black module, in vesicle and synaptic process in the tan module (Table S8). We took red, salmon, tan, and black modules to further analysis, and it is worth noticing that genes of the red module have been enriched in immune response, similar to the previous GO biological processes analysis of the DEGs of chronic pancreatitis, pancreatic cancer, and metastatic pancreatic cancer.

The top20 genes in salmon and red modules significantly overlap with DEGs. The correlation between each gene in a module and the module eigengene defined the eigengene-based connectivity. Since genes with high eigengene-based connectivity can be inferred as playing important roles in this module, we select respectively the top20 genes in four concerned modules to further analysis. We screened the genes from the previous differential analysis. We found that 546 genes were differentially expressed in all three groups: normal-pancreatitis, normal-pancreatic cancer, and normal-metastatic pancreatic cancer. We found that 19 out of 80 genes in four modules overlapped with these 546 genes. There are IFI30, FCER1G, FOLR2, HLA-A, PSMB8, BST2, TNFAIP2, TYROBP, LST1 in the salmon module, CD52, HCLS1, LCK, RAC2, CD27, CD37, CD74 in the red module, HSPA1A, and CLDN5 in the tan module, and FSCN1 in the black module. The heatmap of the expression of these genes shows that their expression does indeed gradually increase in the course of the disease (Fig. 8c).

Most of the top20 genes in the red module showed strong co-expression characteristics, and in the salmon module, PSMB8, PSMB9, PSME2, UBE2L6 do the same. In STRING, the top20 genes in the salmon, red, black, and tan modules respectively get a PPI enrichment P-value of 1.0e − 16, 1.0e − 16, 2.3e − 05, and 0.0494. Since the top20 genes in salmon and red modules showed more correlation than the other two, we verified their co-expression characteristics in STRING and coexpedia (Fig. 8b). The verification result is represented by a graph drawn by Cytoscape.

**Figure 9:**
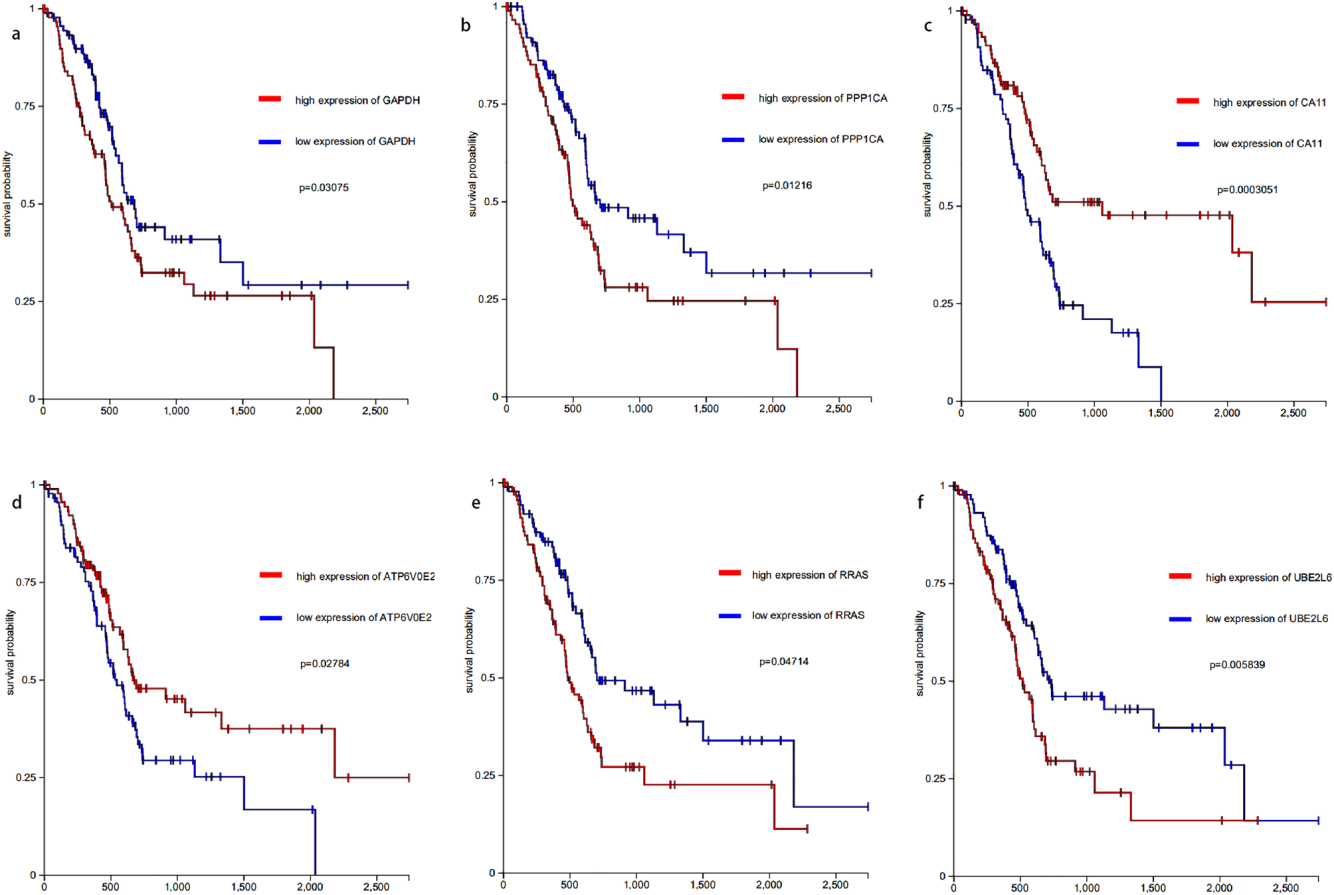
The survival correlation of predicted genes. (**a**) GAPDH; (**b**) PPP1CA; (**c**) CA11; (**d**) ATP6V0E2; (**e**) RRAS; (**f**) UBE2L6.

**Figure 10:**
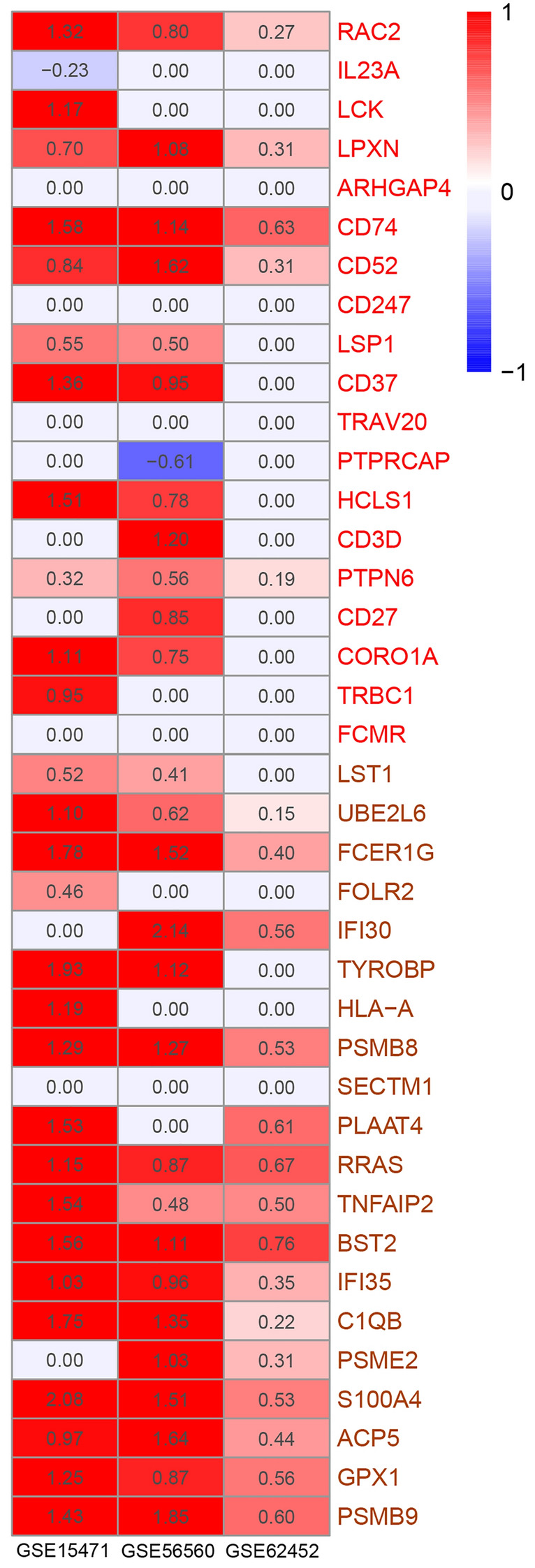
Up-regulation or down-regulation of genes in the data set GSE15471, GSE62452, GSE56560. Upregulated was marked as red ones. Downregulated was marked as blue ones. The white ones with number 0.00 indicate that the gene is not defined as a differentially expressed gene.

We checked the consistency of the analysis results in other data sets. Other data sets proved that the genes in red and salmon modules are upregulated in the pancreatic disease course. We test the top20 genes in the four modules in TCGA. In project TCGA-PAAD, six genes showed survival correlation (Fig. 9a–f). They are PPP1CA and GAPDH in the black module, ATP6V0E2, RRAS, UBE2L6, and CA11 in the salmon module. We also tested our results on data sets containing normal and cancer groups. When adjust P-value of less than 0.05 was considered significant, a total of 40 genes of the red and salmon module showed 29, 20, 27 upregulation, and 1, 0, 1 downregulation in GSE15471, GSE62452, GSE56560, respectively (Fig. 10). It is showed as logFC. Some genes were not defined as differential expression genes in these data sets. Their regulation is valued as 0. Most of the top20 genes in the salmon module showed upregulated in all three external data sets. In the red module, RAC2, LPXN, CD74, CD52, PTPN6, these five genes showed upregulated in all three external data set. In the control of pancreatic cancer and metastatic pancreatic cancer, we did not find a significant difference. We analyzed several data sets GSE42952, TCGA-PAAD, TCGA-LUAD by gene expression differential analysis, and none of them showed upregulation of the gene network. In fact, there were almost no DEGs between cancer and metastatic cancer. We validated these genes at the protein level by HPA database and R package hpar. Expression of these genes in normal tissues is generally lower than that in cancer tissues (Tables S9, 10).

## Discussion

The data set was derived from the entire tissue rather than individual cancer cells. The gene expression of cancer cells and their surrounding environment are included. TME plays an important role in tumor growth and inhibition. Here we find that the gene networks of T cells and interferon are upregulated in stages in the process of pancreatic disease. This is reflected in the red and salmon modules (Fig. 8a, Table S8). And we identified the hub genes of the two gene networks.

The gene network of T cells is upregulated (Fig. 8b). PDAC development is intertwined with multiple types of immunosuppressive cells, including Tregs and MDSCs^8^. Also, PDAC development intertwined with biochemical and physical barriers to T cell infiltration from the surrounding stroma. T cells can have divergent effects on PDAC either by combating cancer growth or by promoting tumor progression via the active induction of immune suppression^26^. In response to this, the red modules identified by WGCNA showed a high correlation with T cells (Table S8). The same goes for the top20 genes in the red module (Fig. 8b). Among these top20 genes CD74, CD27, LCK, CD3D, and CORO1A were proved T cell relatively. CD74 is known as a type II transmembrane glycoprotein that is associated with the major histocompatibility complex class II alpha and beta chains. It is discovered to mediate immune escape^27^. The overexpression of CD74 is a key factor associated with perineural invasion^28–30^. Also, CD74 showed significantly increased expression in a large group of clinical pancreatic adenocarcinomas but were negative in all normal pancreas samples^27^. In glioblastoma, migration inhibitory factor (MIF)-CD74 interaction inhibitor reduced MDSC function and enhanced CD8 T cell activity in the syngeneic mouse model of glioma^31^. Recently antigen-delivery through CD74 was verified as boosting CD8 and CD4 T cell immunity^32^. Activation of the CD27/CD70 axis might have immune suppressive effects. CD27 enhanced survival signal in Tregs and induction of apoptosis of effector T cells^33^. And other genes are thought to be closely related to pancreatic cancer, such as LCK^34^. LCK is critical for T cell development and activation, as it is the first kinase to divert TCR signalling^35^. What's more, LCK, CD247, CD27, CD3D, and CD74 were mentioned as upregulated genes in anti-PDL1 treated mice and downregulated genes in inflammatory breast cancer with PDL1 overexpression^36,37^. RAC is often referred to as the Ras proto-oncogene superfamily^38^. Rac1 and Rac2 control the formation of dendrites in mature dendritic cells. Rac1 and Rac2 control dendritic cells polarized short-range migration toward T cells, and T cell priming^39^. Coronin-1A (CORO1A) is a regulator of actin dynamics important for T cell homeostasis^40^. CORO1A expression in T cells is essential for the activation of autoantigen-specific T cells^41^.

The gene network of the interferon pathway is upregulated (Fig. 8b). Activation of the stimulator of interferon within the tumor microenvironment has been shown to generate an antitumor response. The salmon modules identified by WGCNA relative to type I interferon (Table S8). PSMB8 and PSMB9 can be the alternative subunits to help the constructive proteasome transform to the immune proteasome induced by interferon γ^42^. PSMB8, PSMB9 were identified as potential targets for the diagnosis and therapy of cutaneous squamous-cell carcinoma and melanoma^42,43^.

The regulation of T cells was also detected in pancreatic intraepithelial neoplasia (PanIN), and T cells can promote tumor development through immunosuppression and epithelial-mesenchymal-transition (EMT). In this study, we focused on the progression of pancreatic cancer induced by chronic pancreatitis. There are other diseases that may develop into pancreatic cancer. PanIN is considered a precancerous lesion of pancreatic cancer. PanIN formation is accompanied by a variety of changes to the immune milieu of the pancreas, including an influx of tumor-associated macrophages, MDSCs, and CD4^+^ Tregs. These changes persist and intensify upon progression to malignancy^44,45^. Chronic pancreatitis promotes induction of EMT in premalignant cells of PanIN leading to their dissemination before the detection of a primary PDAC in endogenous mouse model^46^. CD4^+^CD25^−^ T-effector cells and Tregs also contribute to the EMT and invasive phenotype. Elevated levels of TNF-α and IL-6 secreted by T-effector cells account for that^47^. PanIN is an important part of the malignant transformation of pancreatic diseases. In this work, PanIN should be compared with normal state, pancreatitis, pancreatic cancer and metastatic cancer. However, there is no suitable data set for the time being. Data set GSE19650 collects the mRNA expression of the epithelial cells from normal pancreatic ducts, intraductal papillary-mucinous adenoma (IPMA), intraductal papillary-mucinous carcinoma (IPMC), and invasion cancer originating in intraductal papillary-mucinous neoplasm (IPMN). The gene network of T cell regulation mentioned above did not show significant differential expression among IPMA, IPMC and IPMN in GSE19650. We believe that different sample sources have led to this result. Because at least the upregulation of the network of T cell regulation in pancreatic cancer tissues is proved in more other data sets and databases. This difference suggested that the regulation of the network of T cell regulation mainly occurs in the TME and has little to do with tumor cells.

We performed WGCNA and other analyses using relatively conventional methods. But we chose the module from a unique perspective. We chose those modules with continuous changes in correlation coefficients, instead of the modules with the highest correlation. This is a unique part of our research. In fact, if the four consecutive traits of normal, pancreatitis, pancreatic cancer, and metastatic pancreatic cancer are represented by 0, 1, 2, and 3. It is used to represent the gradual high expression of the gene network in the course of the disease. Then the red and salmon modules also have a high correlation with the disease process (Supplementary Fig. S5).

The WGCNA analysis in other studies showed different results. The hub genes of some studies are mostly classified as blue modules^48^. Some are primarily classified in blue modules^49^ or turquoise module^18,50^. And some are scattered in different modules with no obvious slant^51^. This may be due to the differences in samples and analysis focus.

We conducted external verification of our proposed gene network, including RNA level and protein level. In normal and pancreatic cancer controls, the upregulation of our gene network was confirmed. In the control of pancreatic cancer and metastatic pancreatic cancer, we did not find a significant difference. It is possible that when pancreatic cancer transforms into metastatic cancer, it cannot be detected by gene expression differential analysis. The uploaders of the data sets did not provide enough detailed information about the sampling location, so it may also be caused by different sampling locations.

In summary, we studied the transcriptome analysis of pancreatic disease and as a subset of analyses, normal to chronic pancreatitis to PDAC to metastatic disease. The relationship in the immune response and transcription profiles among two different types of pancreatic disease and more specifically in a minor component of pancreatic cancer progression was identified. The network analysis helps to find key genes in pancreatic disease, but we still need experimentally evaluate the function of these genes.

## Supplementary Information

Supplementary Legends.

Supplementary Table S1.

Supplementary Table S2.

Supplementary Table S3.

Supplementary Table S4.

Supplementary Table S5.

Supplementary Table S6.

Supplementary Table S7.

Supplementary Table S8.

Supplementary Table S9.

Supplementary Table S10.

Supplementary Figure S1.

Supplementary Figure S2.

Supplementary Figure S3.

Supplementary Figure S4.

Supplementary Figure S5.

## Abbreviations

DEGs: Differentially expressed genes
EMT: Epithelial-mesenchymal-transition
GO: Gene ontology
IPMA: Intraductal papillary-mucinous adenoma
IPMC: Intraductal papillary-mucinous carcinoma
IPMN: Intraductal papillary-mucinous neoplasm
MDSCs: Myeloid-derived suppressor cells
PanIN: Pancreatic intraepithelial neoplasia
PDAC: Pancreatic ductal adenocarcinoma
SAM: Significance analysis of microarrays
TME: Tumor microenvironment
Tregs: Regulatory T cells
WGCNA: Weighted gene co-expression network analysis
