# Supplementary material for: Transcriptome analysis of the procession from chronic pancreatitis to pancreatic cancer and metastatic pancreatic cancer
Source: Sci Rep. 2021 Feb 9;11:3409. doi: 10.1038/s41598-021-83015-4 (PMC7873308; doi:10.1038/s41598-021-83015-4)
Supplement: Supplementary file 1 — Supplementary Legends. [file 41598_2021_83015_MOESM1_ESM.docx]

**Transcriptome analysis of the procession from chronic pancreatitis to pancreatic cancer** **and metastatic pancreatic cancer**

Jihao Tu^1#^, Zhaohao Huang^2#^, Yin Wang^1^, Meijing Wang^1^, Zukun Yin^3^, Xianglin Mei^4*^, Meiying Li^1*^, Lisha Li^1*^

^1^The Key Laboratory of Pathobiology, Ministry of Education, College of Basic Medical Sciences, Jilin University, Changchun, China

^2^Department of Neurosurgery, The Third Hospital of Jilin University, Changchun 130033, Jilin, China.

^3^Department of Otorhinolaryngology-Head and Neck Surgery, Minda Hospital of Hubei Minzu University, Enshi, China.

^4^Department of Pathology, The Second Hospital of Jilin University, Changchun 130041, Jilin, China.

^#^ These authors contributed equally to this study

* Corresponding authors: Lisha Li (E‑mail: lilisha@jlu.edu.cn), Meiying Li (E‑mail: limeiying@jlu.edu.cn), Xianglin Mei (E‑mail: meixianglin105@163.com).

Table S1 all the genes expression value in 36 samples

Table S2 DEGs in chronic pancreatitis by fold change and all the enriched analysis

Table S3 DEGs in pancreatic cancer by fold change and all the enriched analysis

Table S4 DEGs in metastatic pancreatic cancer by fold change and all the enriched analysis

Table S5 Significant DEGs by SAM and all the enriched analysis

Table S6 The network analysis of string network of significant DEGs by SAM

Table S7 MCODE analysis of string network of significant DEGs by SAM

Table S8 The merged colors and number of genes from the obtained modules in E- EMBL-6

Table S9 Top 5 Gene Ontology Biological Processes of modules of co-expression modules

Figure S1 A total of 36 samples cluster visualization

Figure S2 Selecting the property soft threshold power, β value

Figure S3 Module visualization of genes

Figure S4 Visualization of correlations between modules
