# Supplementary figures and images for: Transcriptome analysis of the procession from chronic pancreatitis to pancreatic cancer and metastatic pancreatic cancer
Source: Sci Rep. 2021 Feb 9;11:3409. doi: 10.1038/s41598-021-83015-4 (PMC7873308; doi:10.1038/s41598-021-83015-4)

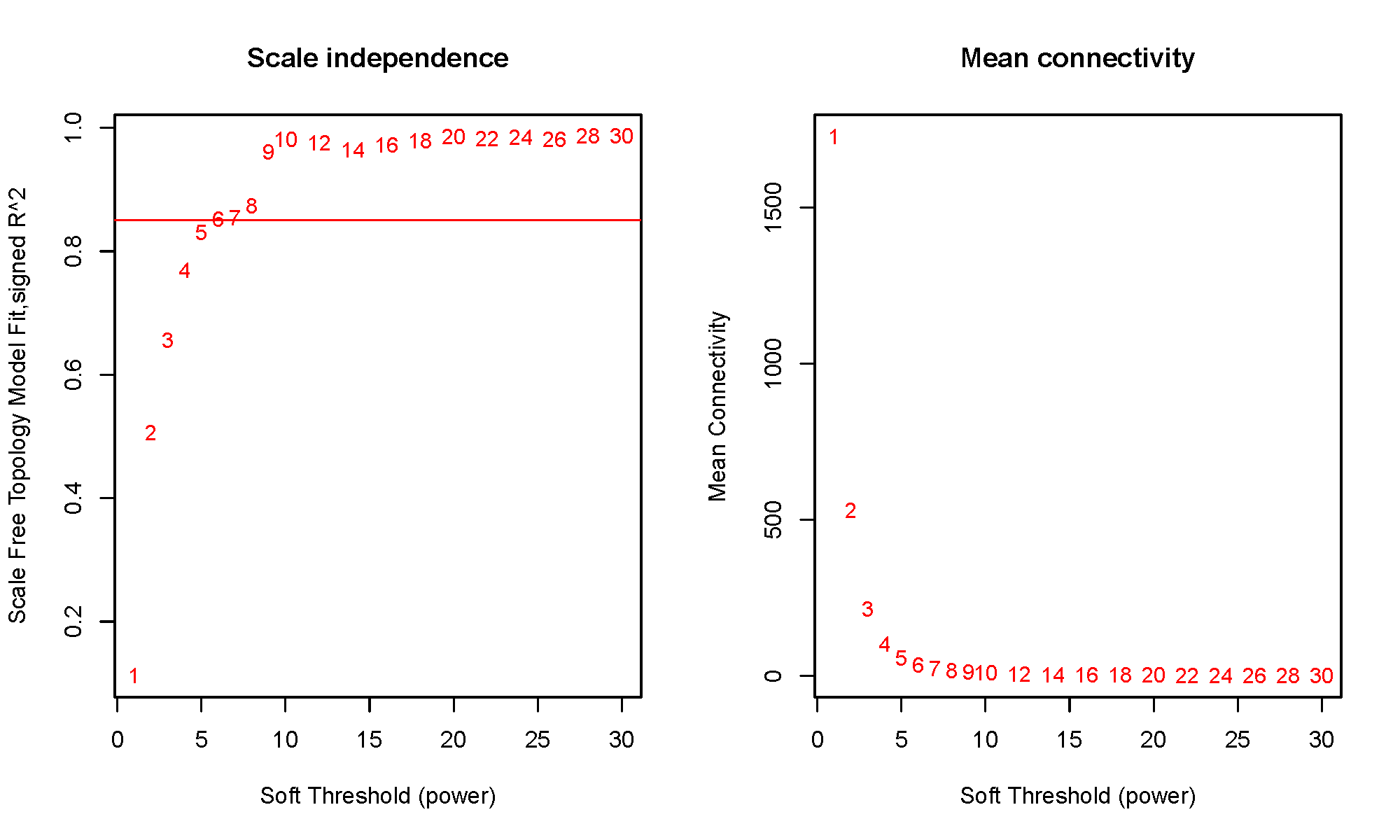

Supplement: Supplementary file 13 — Supplementary Figure S2. [file 41598_2021_83015_MOESM13_ESM.tif]

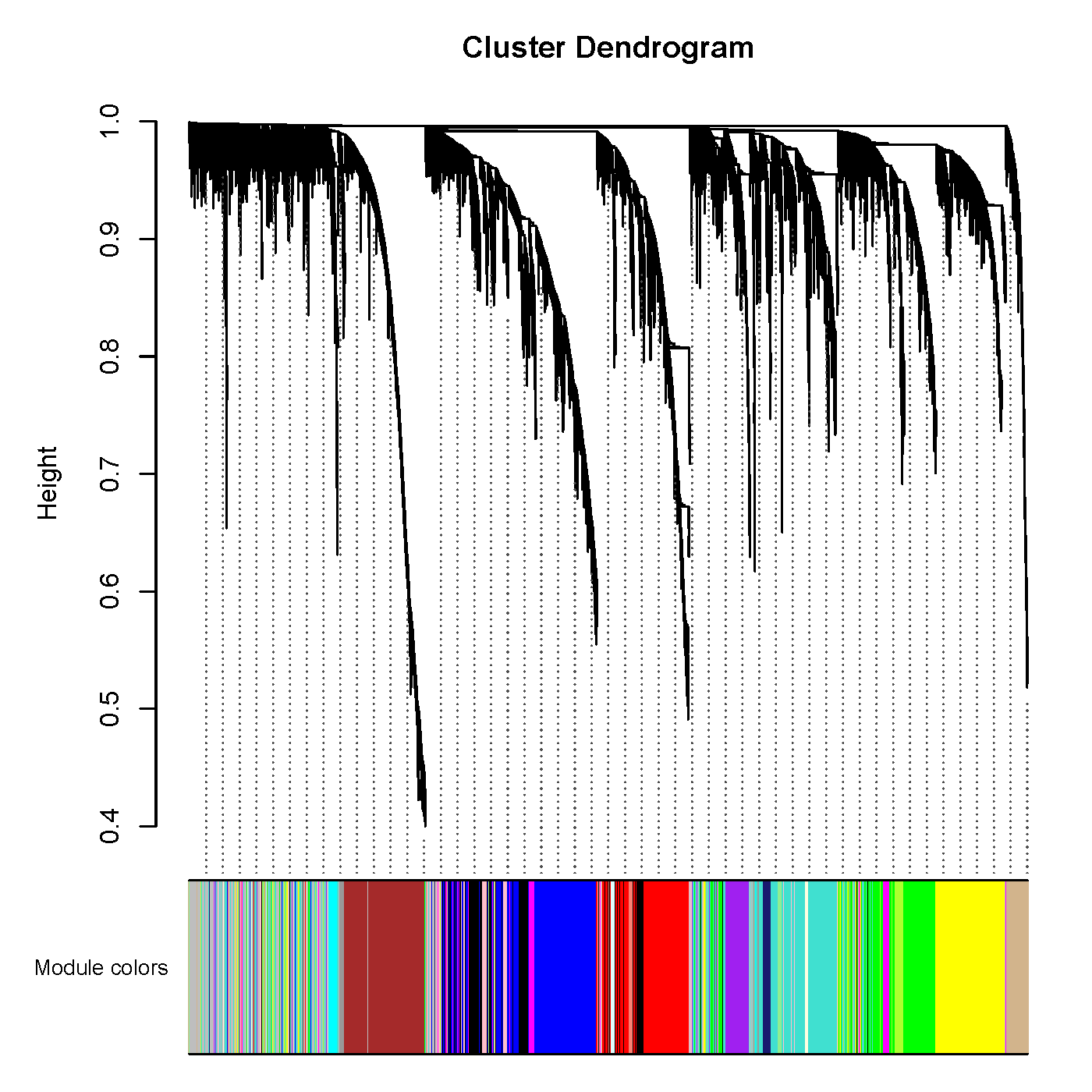

Supplement: Supplementary file 14 — Supplementary Figure S3. [file 41598_2021_83015_MOESM14_ESM.tif]

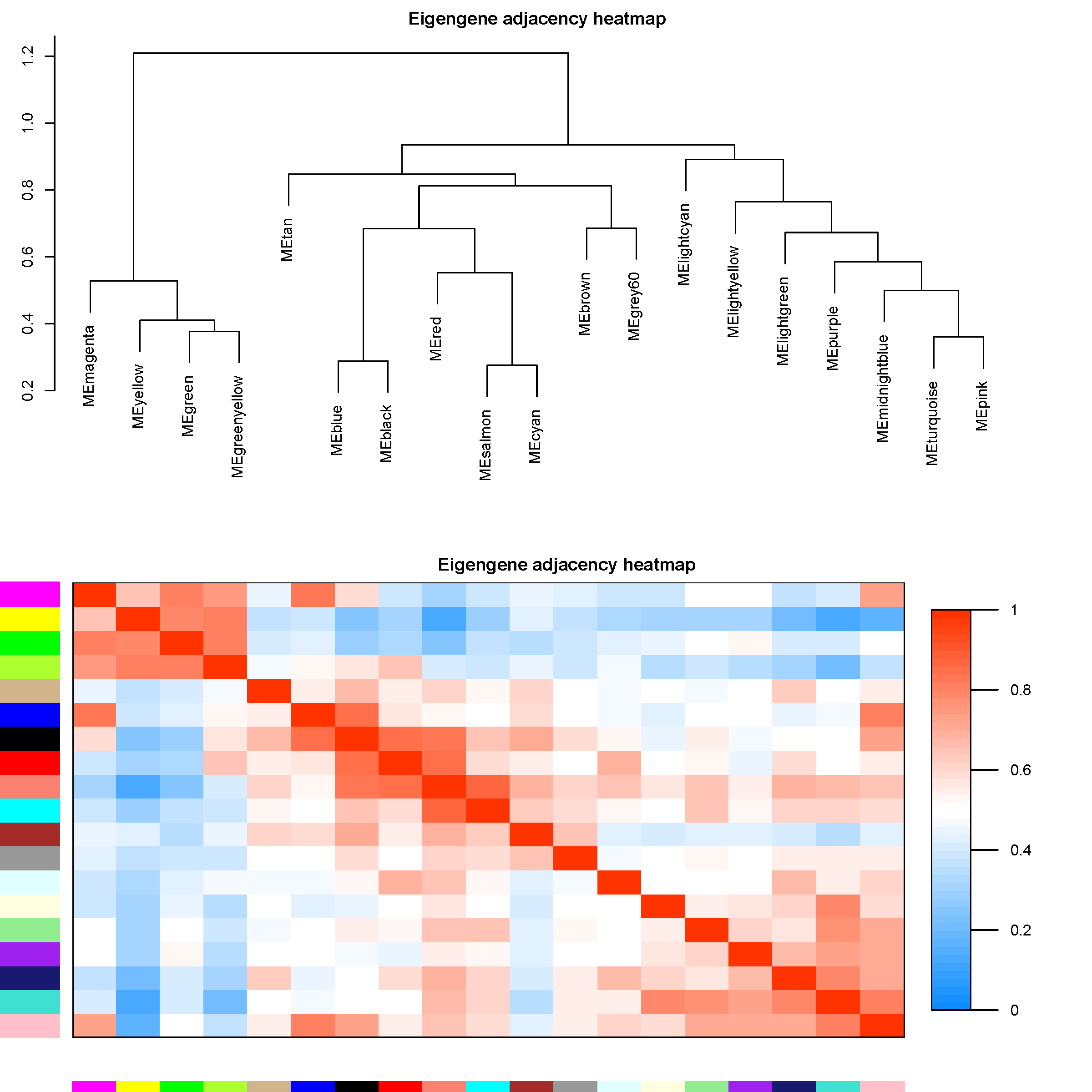

Supplement: Supplementary file 15 — Supplementary Figure S4. [file 41598_2021_83015_MOESM15_ESM.tif]

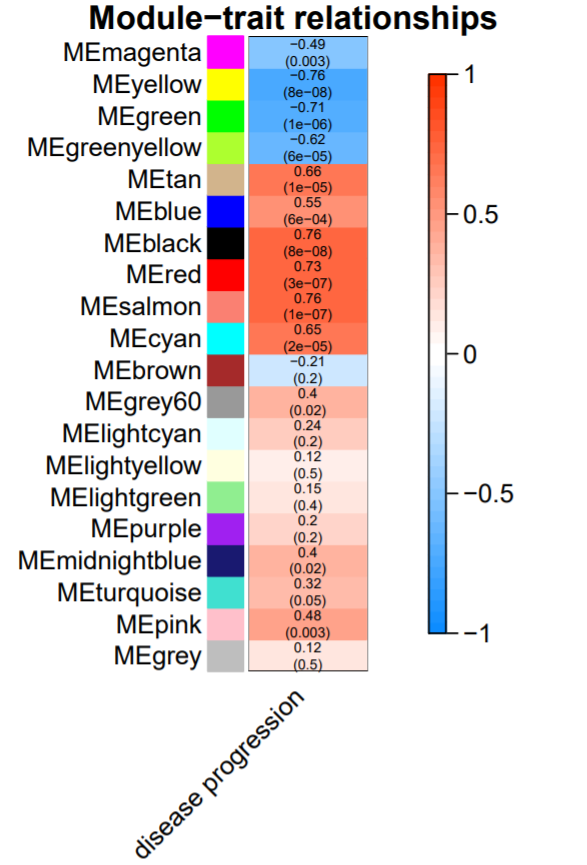

Supplement: Supplementary file 16 — Supplementary Figure S5. [file 41598_2021_83015_MOESM16_ESM.tif]
